# Does neighbourhood crime mediate the relationship between neighbourhood SES and birth outcomes?

**DOI:** 10.1093/eurpub/ckac130.041

**Published:** 2022-10-25

**Authors:** L Burgos Ochoa, MJ Bijlsma, EAP Steegers, JV Been, LCM Bertens

**Affiliations:** 1 Obstetrics and Gyneacology, Erasmus MC, Rotterdam, Netherlands; 2 Pharmacotherapy, Epidemiology and Economics, University of Groningen, Groningen, Netherlands; 3 Division of Neonatology, Paediatrics, Erasmus MC, Rotterdam, Netherlands; 4 Department of Public Health, Erasmus MC, Rotterdam, Netherlands

## Abstract

**Background:**

Previous studies have consistently found that women living in low socioeconomic status (SES) neighbourhoods are at higher risk of experiencing adverse birth outcomes compared to women from high SES areas. However, the mechanisms through which neighbourhood SES might influence health at birth remain poorly understood. One of the proposed pathways is the exposure to higher crime rates. The aim of this study is to investigate whether neighbourhood crime mediates the relationship between neighbourhood SES and birth outcomes.

**Methods:**

A retrospective cohort study including over 1.3 million singleton births occurred in the Netherlands between 2010 and 2017. Individual-level data from the Dutch perinatal registry was linked to quintiles of neighbourhood SES scores and neighbourhood-level crime rates. Using the mediational g-formula, we estimated the total effect, natural direct effect, and natural indirect effect of neighbourhood SES on birth outcomes: small-for-gestational-age (SGA), low birth weight, and preterm birth. The neighbourhood SES intervention settings correspond with a hypothetical improvement in neighbourhood SES from the lowest to the highest quintile.

**Results:**

The hypothetical improvement in neighbourhood SES resulted in a 6.6% (CI = 5.6%; 7.5%) relative reduction in the proportion of SGA births, an 8.9% (CI = 7.6%; 10.3%) reduction in the proportion of low birth weight, and a 5.1% (CI = 4.0%; 6.1%) decrease of preterm birth. Neighbourhood crime accounted for 29.0% (CI = 25.1%; 32.8%) of the total effect of neighbourhood SES on SGA, and for 8.6% (CI = 5.1%; 11.6%) of the total effect on low birth weight. For preterm birth, we found no evidence of mediation by neighbourhood crime.

**Conclusions:**

Neighbourhood crime mediates the association between neighbourhood SES and key adverse birth outcomes. Interventions targeted at lowering neighbourhood crime rates could improve birth outcomes in disadvantaged areas.

**Key messages:**

- Neighbourhood crime mediates the association between neighbourhood SES and adverse birth outcomes in the Netherlands.
- Results from this study can provide guidance to neighbourhood-level strategies aiming at improving health at birth and reducing health inequalities.

